# Post-translational regulation of metabolism in fumarate hydratase deficient cancer cells

**DOI:** 10.1016/j.ymben.2017.11.011

**Published:** 2018-01

**Authors:** Emanuel Gonçalves, Marco Sciacovelli, Ana S.H. Costa, Maxine Gia Binh Tran, Timothy Isaac Johnson, Daniel Machado, Christian Frezza, Julio Saez-Rodriguez

**Affiliations:** a European Molecular Biology Laboratory, European Bioinformatics Institute, EMBL-EBI, Wellcome Genome Campus, Cambridge CB10 1SD, UK; b Medical Research Council Cancer Unit, University of Cambridge, Cambridge CB2 0XZ, UK; c European Molecular Biology Laboratory, EMBL, Heidelberg, Germany; d UCL Division of Surgery and Interventional Science, Specialist Center for Kidney Cancer, Royal Free Hospital, Pond Street, London NW3 2QG, UK; e RWTH Aachen University, Faculty of Medicine, Joint Research Center for Computational Biomedicine, Aachen, Germany; f Centre of Biological Engineering, University of Minho, Braga, Portugal

**Keywords:** Metabolism, Cancer, Phosphoproteomics, Modelling

## Abstract

Deregulated signal transduction and energy metabolism are hallmarks of cancer and both play a fundamental role in tumorigenesis. While it is increasingly recognised that signalling and metabolism are highly interconnected, the underpinning mechanisms of their co-regulation are still largely unknown. Here we designed and acquired proteomics, phosphoproteomics, and metabolomics experiments in fumarate hydratase (FH) deficient cells and developed a computational modelling approach to identify putative regulatory phosphorylation-sites of metabolic enzymes. We identified previously reported functionally relevant phosphosites and potentially novel regulatory residues in enzymes of the central carbon metabolism. In particular, we showed that pyruvate dehydrogenase (PDHA1) enzymatic activity is inhibited by increased phosphorylation in FH-deficient cells, restricting carbon entry from glucose to the tricarboxylic acid cycle. Moreover, we confirmed PDHA1 phosphorylation in human FH-deficient tumours. Our work provides a novel approach to investigate how post-translational modifications of enzymes regulate metabolism and could have important implications for understanding the metabolic transformation of FH-deficient cancers with potential clinical applications.

## Highlights

• Fumarate hydratase (FH) deficiency leads to profound phosphorylation changes.
• Genome-scale modelling identified regulatory phosphosites in metabolic enzymes.
• Pyruvate dehydrogenase is inhibited by phosphorylation in FH deficient cancer cells.
• HLRCC tumours have increased PDH phosphorylation compared to normal kidney tissue.

## 1 Introduction

Cancer is thought to arise from an abnormal accumulation of somatic mutations in the genome that drive complex and profound alterations of the cellular phenotype (Stratton et al., 2009). Among these changes, dysregulated energy metabolism is gaining importance as a hallmark of cancer (Hanahan and Weinberg, 2011). Although some recent work elucidated the genetic underpinning of these metabolic changes (Gaude and Frezza, 2016, Hu et al., 2013), whether cancer metabolism is tuned via post-translational changes is still largely unknown. In yeast, several studies have shown that signalling has a broad importance in regulating the activity of metabolic enzymes involved in central carbon metabolism and other peripheral pathways (Gonçalves et al., 2017, Oliveira et al., 2015, Raguz Nakic et al., 2016). By contrast, regulatory phosphorylation of metabolic enzymes in human cells remains largely uncharacterised.

A particularly well-studied metabolic alteration in cancer is driven by mutations of the metabolic enzyme fumarate hydratase (FH). These mutations cause Hereditary Leiomyomatosis and Renal Cell Cancer (HLRCC) tumours, a cancer syndrome characterised by benign tumours of the skin and uterus, and a very severe and aggressive form of renal cancer (Tomlinson et al., 2002). FH catalyses the conversion of fumarate to malate, a metabolic reaction that takes part in the tricarboxylic acid cycle (TCA cycle). FH mutations lead to the impairment of the catalytic activity of the enzyme and thereby to the accumulation of its substrate, fumarate, and consequently to profound metabolic changes that we and others have extensively characterised (Isaacs et al., 2005, Sciacovelli et al., 2016, Tomlinson et al., 2002). Yet, how these metabolic changes are orchestrated by signalling processes has not been investigated.

**Fig. 1:**
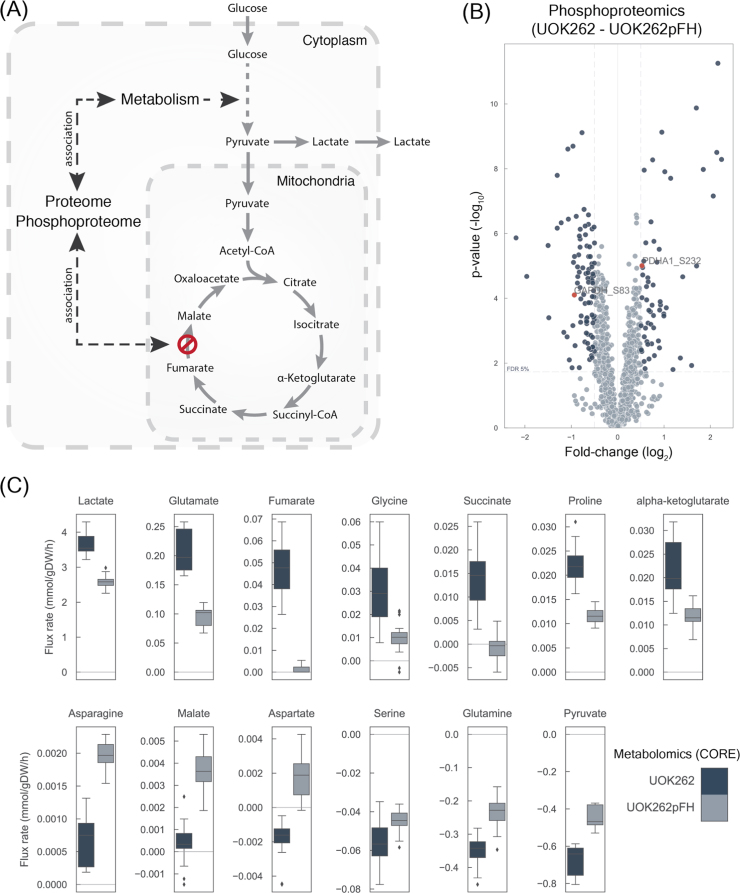
Molecular characterisation of HLRCC derived UOK262 and UOK262pFH cell lines. A) Diagram depicting the potential molecular implication of fumarate hydratase deletion in the proteome and phosphoproteome and subsequent regulatory implications in metabolism. B) Differential phosphoproteomics analysis. C) Consumption and release (CORE) metabolomics experiments quantifying exchange rates (mmol/gDW/h). All the metabolite rates shown are significantly different (FDR < 5%) between UOK262 and UOK262pFH cells.

Here, we performed an integrative analysis to investigate at a genome-scale level the regulatory interactions between metabolism and signalling using cell lines derived from an HLRCC tumor, UOK262, and the FH reconstituted counterpart, UOK262pFH, which we previously generated (Frezza et al., 2011). In particular, we characterised signalling and metabolic changes in HLRCC cell lines by designing and acquiring phosphoproteomics, proteomics and metabolomics measurements (Fig. 1). These data-sets allowed us to study the molecular adaptations of signalling and metabolism driven by the loss-of-function of FH in HLRCC using a computational framework that integrates phosphoproteomics with *in silico* estimated metabolic flux rates. Pairing the metabolomics modelling with phosphoproteomics measurements allowed us to identify putative-regulatory phosphorylation-sites in metabolic enzymes involved, mostly, in the central carbon metabolism. Notably, we experimentally validated that phosphorylation of pyruvate dehydrogenase E1 component subunit alpha (PDHA1) regulates glucose fluxes into the TCA cycle in FH-deficient cells. In summary, we present a novel computational and experimental approach to systematically identify putative regulatory phosphorylation-sites in metabolic enzymes. This approach could reveal novel regulatory networks in the metabolic transformation of cancer, with important implications for cancer therapy.

## 2 Results

### 2.1 Characterisation of the phosphoproteome of human FH-deficient cells

We started our investigation by comparing the proteome of human FH-deficient UOK262 cells and their FH-complemented counterpart, which we previously generated (Frezza et al., 2011, Sciacovelli et al., 2016) (see Methods). Proteomics experiment covered a total of 1468 unique proteins (Supplementary Table 1) and, in agreement with FH mutation, FH was underexpressed in UOK262 cell lines. Reproducibility of the measurements was assessed with unsupervised hierarchical clustering where replicates showed higher correlation coefficients than all the pairwise comparisons (Supplementary Fig. 1). Proteomics showed agreement with the RNA-seq transcriptomics measurements available for the same cell lines (spearman's rho (r) of 0.43, *p-value* = 1.7e−63) (Sciacovelli et al., 2016) (Supplementary Fig. 2A). Some proteins displayed a disagreement between the protein abundance and the transcript expression, reflecting different types of regulatory mechanisms occurring at post-transcriptional and post-translational levels, consistent with previous reports (Sciacovelli et al., 2016).

To study post-translational modifications by phosphorylation we characterised the phosphoproteome of these cell lines. In total, we measured 1360 unique single phosphorylated phosphosites, mapping to 812 unique proteins (Fig. 1B) (Supplementary Table 1). Similarly, to the proteomics measurements, VIM also showed a significant increase in phosphorylation in the UOK262 cell lines, although these changes are associated with the increase in protein abundance. Metabolic enzymes displayed significant changes between UOK262 and UOK262pFH, in particular PDHA1 and GAPDH (Fig. 1B). Specifically, 56% (23/41) of the phosphosites in metabolic enzymes display significant changes (FDR < 5%) (Table 1). This supports the idea that metabolic enzymes are regulated by phosphorylation in FH-deficient UOK262 cell lines.

**Table 1:** Metabolic enzymes differentially phosphorylated sites.

**Enzyme**	**Phosphosite**	**Fold-change**	**p-value**	**FDR**
GAPDH	S83	− 0.93	7.9E−05	9.3E−04
HMGCS1	S495	− 0.81	7.3E−07	3.3E−05
CTPS1	S575	− 0.70	1.4E−04	1.4E−03
MTMR3	S613	− 0.52	5.8E−05	7.2E−04
CTPS1	S574	− 0.47	4.5E−05	6.2E−04
PGK1	S203	− 0.40	5.2E−05	6.7E−04
DPYSL3	T509	− 0.39	5.5E−05	6.9E−04
PGM1	S117	− 0.39	1.8E−04	1.6E−03
IMPDH1	S160	− 0.37	3.3E−03	1.3E−02
IMPDH2	S160	− 0.37	3.3E−03	1.3E−02
PIK3C2A	S884	− 0.36	5.2E−04	3.4E−03
DPYSL3	S522	− 0.36	8.1E−05	9.3E−04
PI4KB	S428	− 0.34	4.4E−04	3.0E−03
PGM3	T62	− 0.16	7.9E−03	2.6E−02
PCYT1A	S315	0.37	1.9E−04	1.7E−03
PCYT1B	S315	0.37	1.9E−04	1.7E−03
BCKDHA	S347	0.41	1.5E−03	7.6E−03
NAA10	S205	0.46	4.3E−03	1.6E−02
RRM2	S20	0.47	1.8E−04	1.6E−03
GUCY1B2	S150	0.52	4.4E−03	1.6E−02
PDHA1	S232	0.52	9.8E−06	2.3E−04
CMPK1	S180	0.67	7.4E−03	2.5E−02
BCKDHA	S337	0.73	2.5E−03	1.1E−02

### 2.2 Genome-scale metabolic modelling

**Fig. 2:**
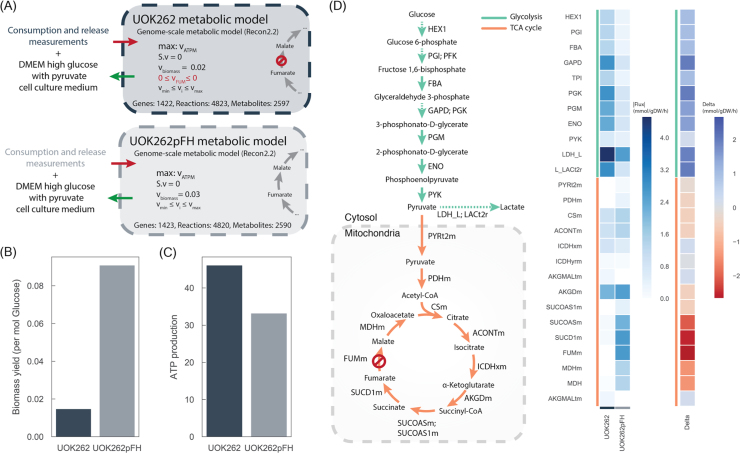
Genome-scale metabolic modelling of UOK262 cell lines. A) Diagram depicting the different constraints used in Recon 2.2 to obtain the condition-specific, UOK262 and UOK262pFH, metabolic models. B) Biomass yield per mol of glucose intake calculated from experimental measurements. C) Maximum ATP production of both models. D) Flux distributions of glycolysis and TCA cycle pathways reactions estimated by maximising ATP production using pFBA.

To investigate how phosphorylation of metabolic enzymes could regulate metabolism, we computed the intracellular metabolic fluxes of FH-deficient cells using genome-scale reconstruction of human metabolism (Duarte et al., 2007, Swainston et al., 2016, Thiele et al., 2013). To this end, we capitalised on a recently generated version of the human genome-scale metabolic model (Swainston et al., 2016) and constrained it using consumption/release (CORE) measurements (Fig. 1C), growth rates (Supplementary Table 1), and the FH loss status, to generate specific models for UOK262 and UOK262pFH cell lines separately (Fig. 2A) (see Methods). In particular, FH loss in UOK262 cells was modelled by limiting the flux rate of its catalysed reactions to zero, while in the UOK262pFH cells they remained unaltered. CORE rates were measured using liquid chromatography mass spectrometry analysis of spent media (Fig. 1C) (Supplementary Table 1). An unsupervised hierarchical clustering showed that UOK262 and UOK262pFH CORE rates clustered separately (Supplementary Fig. 1). UOK262 cells displayed increased lactate secretion (Fig. 1C), and, while not significant at an FDR 5% (FDR < 10%), they also showed increase of glucose consumption (Supplementary Table 1), in line with aerobic glycolysis in FH-deficient cells (Frezza et al., 2011).

Parsimonious FBA (pFBA) (Lewis et al., 2010) was then used to simulate the two metabolic models using ATP production as the objective function (Supplementary Table 2). ATP production was represented by the ATP maintenance reaction (ATPM) (see Methods). UOK262 cells showed decreased biomass yield, suggesting that the impairment of the mitochondrial function by FH mutation and the increased levels of glucose intake does not lead to augmented growth rate (Fig. 2B). The maximisation of the ATPM predicted increased levels of energy production of UOK262 cells (Fig. 2C). Of note, the total amount of ATP production in UOK262 was greater than what would be expected by glucose uptake alone, suggesting that other carbon sources are utilised by these cells. This is supported by the measured increased uptake of glutamine.

Glycolytic reactions displayed increased fluxes and increased levels of lactate secretion in UOK262 cells (Fig. 2D), in line with previous observations (Frezza et al., 2011). Interestingly, the models also captured the impaired mitochondrial activity of UOK262 cells. Pyruvate dehydrogenase (PDHm) reaction shows decreased intake of pyruvate into the mitochondria and FH inactivation leads to decreased metabolic activity of several reactions in the TCA cycle, e.g. MDHm, SUCD1m. While these models do not account for intracellular accumulation and depletion of metabolites, these results are in line with the accumulation of fumarate, succinate, and succinyl-coa, as aKGDm displays consistent activity. Of note, increase of fumarate is a biochemical feature of FH-deficient cells (Frezza et al., 2011). In summary, these models recapitulate several known metabolic phenotypes of these cell lines and therefore offer the possibility to explore at a genome-scale level the metabolic adaptations of UOK262 to the reactivation of FH.

### 2.3 Post-translational and post-transcriptional regulation of metabolism

**Fig. 3:**
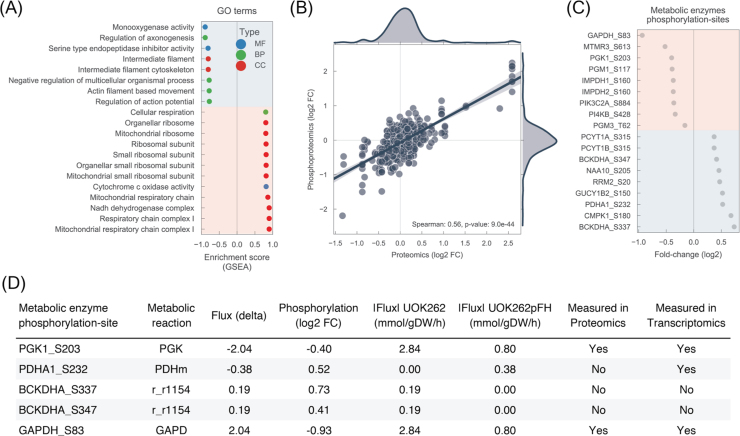
Post-translational regulation of metabolism in FH-deficient cells. A) Top significantly enriched GO terms found in the proteomics data-set (UOK262 - UOK262pFH). Red background denotes GO terms that are down-regulated and blue background denotes up-regulated. MF: molecular function; CC: cellular component; BP: biological process. B) Correlation between proteomics and phosphoproteomics measurements. C) Phosphorylation-sites located in metabolic enzymes for which the protein abundance is either not changing significantly or it was not measured. D) List of putative regulatory phosphorylation-sites in metabolic enzymes. Candidates were selected from C) and sorted by the metabolic flux change (flux delta). The top 5 absolute metabolic flux changes are shown. (For interpretation of the references to color in this figure legend, the reader is referred to the web version of this article.)

Having evaluated the predictive capacity of the metabolic models, we then used the *in silico* metabolic fluxes together with the proteomics and phosphoproteomics data-sets to explore potential regulatory mechanisms of metabolism. An exploratory enrichment analysis (Subramanian et al., 2005) of the proteomics revealed several Gene Ontology (GO) terms (The Gene Ontology Consortium, 2015) significantly enriched in these cells (Fig. 3A) (Supplementary Table 2). In particular, processes involving cellular filament and cytoskeleton were identified to be significantly up-regulated in UOK262. This result is consistent with the increased motility of UOK262 cells, which has also been associated with epithelial to mesenchymal transition (EMT) (Sciacovelli et al., 2016). Several GO terms related with mitochondrial processes, such as respiratory chain complexes, were down-regulated, consistently with the metabolic model predictions and previously observed decreased mitochondrial activity (Sciacovelli et al., 2016, Tyrakis et al. 2017). We then assessed if changes in protein abundance of metabolic enzymes could be related with metabolic flux changes predicted by the model (Supplementary Fig. 2B). Correlation analysis showed no significant relationship (Spearman's r = 0.12, p-value = 5.21e−01), suggesting that enzyme abundance is insufficient to determine metabolic fluxes, which is consistent with the limited success of previous approaches to interpret metabolism using transcriptomics and proteomics data (Machado and Herrgård, 2014). Nevertheless, several metabolic pathways displayed a consistent profile at protein and flux level, for example TCA cycle decreased protein abundance and decreased metabolic flux in UOK262. Intriguingly, glutamate metabolism shows an increase in the abundance of metabolic enzymes and decrease in metabolic flux of the whole pathway in UOK262 (Supplementary Fig. 2B), which was not in agreement with the measured increase in glutamate secretion and glutamine intake (Fig. 1C). We validated this unexpected finding performing a separate ^13^C-glutamine labelling experiment and found that indeed, whilst these cells do not accumulate glutamate (Zheng et al., 2013), they release glutamate in a time-dependent fashion, and glutamate is predominantly generated by glutamine (Supplementary Fig. 2C, D). The poor correlation between protein abundance and metabolic fluxes suggested a potential regulatory role of post-translational changes in these metabolic enzymes.

To find potential regulatory phosphorylation sites of metabolic enzymes we first assessed the correlation between phosphorylation levels and the respective abundance of the protein. As expected, phosphosites fold-changes were tightly correlated with protein abundance (spearman's r = 0.56, p-value = 9.0e−44) (Fig. 3B). To focus on phosphorylation changes that are independent of the protein abundance we only considered residues that change significantly in phosphorylation but not in abundance. This process allowed us to obtain a list of 18 phosphorylation-sites in metabolic enzymes that show significant changes in phosphorylation in FH-deficient cells (Fig. 3C).

**Fig. 4:**
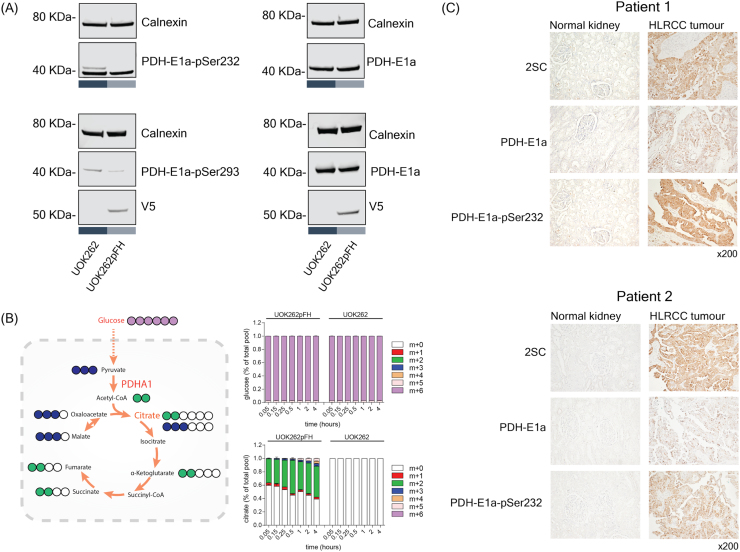
Experimental validation of PDHA1 phosphorylation regulation in FH-deficient cells. A) Western Blot of PDHA1 protein abundance (PDH-E1a) and PDHA1 S232 (PDH-E1a-pSer232) and S293 (PDH-E1a-pSer293) phosphorylation. Calnexin was used as loading control and V5 to stain re-expressed V5-FH-wt in UOK262pFH. B) ^13^C-Glucose labelling experiment tracking the uptake of glucose into the mitochondria via PDHA1. C) Immunohistochemistry of HLRCC tumours and corresponding adjacent normal kidney tissue stained for PDHA1 and PDHA1 S232 phosphorylation and 2-succinic-cysteine (2SC).

Subsequently, we enquired which of these differentially phosphorylated sites are more likely to have a functional impact in the metabolic enzyme. To that end, we resorted to the *in silico* estimated fluxes to rank the metabolic enzymes according to the flux changes, UOK262 vs UOK262pFH, on their catalysed reactions. In particular, we used the genomic annotation in the metabolic model to map the selected phosphosites in the metabolic enzymes to the reactions they catalyse, covering 20 phosphosite-reaction interactions (Supplementary Table 4) (Fig. 3D). With our analysis, we recapitulated 3 previously reported regulatory residues in PhosphositePlus (Hornbeck et al., 2015), i.e. PDHA1_S232, PGK1_S203 and RRM2_S20. Among the top changing reactions with matched phosphorylation changes is PDHA1, where reaction PDHm shows decreased metabolic flux and increased phosphorylation in S232. This result is consistent with current literature that shows that increased phosphorylation in any of the serine residues in positions 232, 293 and 300 of PDHA1 inactivates the enzyme, and its function is only restored when these residues have been dephosphorylated (Kato et al., 2008, Korotchkina and Patel, 2001, Seifert et al., 2007). While PDHA1 phosphorylation was detected in the phosphoproteomics, its total protein abundance was not detected in the proteomics data-set. This is likely due to inherent technical limitations of mass-spectrometry experiments to detect the full expressed proteome. To further validate our predictions, we first confirmed PDHA1 abundance by Western Blotting (WB), and showed that there are no significant changes between UOK262 and UOK262pFH (Fig. 4A). Also, we confirmed, by WB, that S232 phosphorylation is significantly increased in FH-deficient cells, and despite no other PDHA1 phosphorylation sites were measured we also validated that S293 shows similar significant increase in phosphorylation (Fig. 4A).

Next, we validated the predicted inactivation of PDHm by measuring the conversion of glucose-derived pyruvate to citrate, a two-step reaction that involves PDH-mediated conversion of pyruvate to acetyl-CoA and the condensation of acetyl-CoA with oxaloacetate to generate citrate. To this aim, cells were incubated with ^13^C_6_-glucose and the isotopologue distribution of pyruvate and citrate was analysed by LC-MS (schematic in Fig. 4B). Whilst glucose uptake is increased in FH-deficient cells (Yang et al., 2013) (Supplementary Fig. 2D), the incorporation of glucose-derived molecules into citrate is significantly reduced in FH-deficient cells, consistent with the inhibition of PDH activity and the prediction of the model based on phosphoproteomics data (Fig. 3C). Lastly, we explored the relevance of our finding in two independent human tumour samples, and consistently with our predictions HLRCC tumours display increased abundance of PDHA1 S232 phosphorylation compared to adjacent normal kidney tissue (Fig. 4C).

## 3 Discussion

Metabolism deregulation is a hallmark of cancer (Hanahan and Weinberg, 2011, Hanahan and Weinberg, 2000) however how these changes are orchestrated is still unclear. Post-translational modifications of metabolic enzymes, including phosphorylation, are emerging as important regulatory checkpoints for fine tuning metabolic pathways. Yet, understanding how these changes control metabolism at a system level requires an integrative perspective. Here we acquired and analysed data characterizing the proteome, phosphoproteome, and metabolome of cell lines lacking FH, to investigate post-translational mechanisms of regulation of their metabolism.

Since only a fraction of the phosphorylation-sites across the proteome have a significant biological role (Beltrao et al., 2012) and as few as 20% are mapped to kinases or phosphatases (Dinkel et al., 2011, Hornbeck et al., 2015), it is important to asses the functional impact of phosphorylation changes. In this work, we provided evidence that combining phosphoproteomics data with measurements of fluxes of metabolic enzymes enables an accurate prediction of how enzyme activity can be regulated by post-translational modifications in human cancer cells. To estimate intracellular metabolic fluxes, we took advantage of genome-scale metabolic models constrained with quantitative experimental data of CORE rates of metabolites. Accurate quantification of the metabolic rates was important to rule out possible confounding effects, such as cell number and cell size, and to robustly estimate the metabolic differences between the two cell lines. The *in silico* estimated metabolic fluxes recapitulated several biological phenotypes of the UOK262 cell lines, for example, increased glycolytic flux, decreased mitochondrial respiratory function and increased lactate secretion (Sciacovelli et al., 2016). In general, metabolic pathway fluxes were not associated with protein abundance, in agreement with results in microorganisms that showed that transcriptional profile is a poor predictor of metabolism (Machado and Herrgård, 2014). These results emphasise the importance to take in consideration other sources of regulation, in particular post-translational modifications such as phosphorylation.

Using metabolic fluxes as readouts of functional activity of metabolic enzymes, we performed a systematic identification of putative regulatory phosphosites of metabolic enzymes by matching phosphorylation changes in the enzymes residues with the flux changes of the catalysed reactions. Phosphorylation changes displayed strong correlation with protein abundance, and we therefore focused on metabolic enzymes that did not have measured significant protein abundance changes. From our list of putative regulatory phosphosites we recapitulated three, PDHA1_S232, PGK1_S203 and RRM2_S20, that were previously reported in PhosphositePlus (Hornbeck et al., 2015). In particular, we explored the potential regulatory implication of S232 in FH-deficient cells. Resorting to glucose labelling experiments we validated the prediction that increased phosphorylation is accompanied by a decreased flux of glucose into the mitochondria. These findings show the functional role of PDHA1 phosphorylation in the regulation of metabolism, and could have important implications in broad cellular phenotypes such as energy production and cell growth. Notably, we showed that increased phosphorylation of PDHA1 S232 is also present in human tumours, reinforcing the importance of this finding and the usefulness of integrated ohmics approaches to reveal molecular changes with potential impact for clinical outcomes. PDHA1 functional phosphosites are regulated by PDK kinases and PDPK phosphatases (Kato et al., 2008, Korotchkina and Patel, 2001, Seifert et al., 2007). While dysregulation of these proteins can provide mechanistic insight into the regulation of PDHA1, further work would need to be carried out, such as perturbation dynamic phosphoproteomics experiments, to validate the regulatory role of PDK and PDPK and of other potential upstream kinases and signalling pathways involved in FH-deficient cells. Another potential regulatory phosphosite is S83 in glyceraldehyde-3-phosphate dehydrogenase (GAPDH) for which our measurements show a significant decrease in phosphorylation and increase in metabolic flux. While further experimental evidence is required to confirm these findings, this hypothesis can potentially provide insights into the inactivation of GAPDH to re-route glycolysis flux into pentose phosphate pathway in response to potential oxidative stress (Grant, 2008) a phenomenon that has been reported in FH deficient cells (Sourbier et al., 2014).

In summary, this work provides for the first time a genome-scale study of the regulatory implications of post-translational modifications in the metabolism of FH-deficient cancer cells. Specifically, we exemplify the utility of our approach to identify in a systematic manner potential regulatory phosphorylation residues in metabolic enzymes, which can help to shed light into the complex regulation of cancer metabolism. This approach is also generally applicable to the study of other types of post-translational modifications that fall on metabolic enzymes, for example acetylation and succination, a modification caused by increased fumarate in FH-deficient cells (Yang et al., 2014). Pairing recent studies that have characterised the phosphoproteome of several hundreds of tumour samples (Mertins et al., 2016, Zhang et al., 2016) with metabolomics measurements will potentiate the discovery of novel therapies that exploit ubiquitous features of cancer metabolism.

## 4 Methods

### 4.1 UOK262 and UOK262pFH cells growth

Human FH-mutant UOK262 and FH-reconstituted UOK262pFH cells were obtained as previously described (Frezza et al., 2011, Sciacovelli et al., 2016). All cells were grown in DMEM (Gibco 41966-029) supplemented with 10% heat inactivated FBS (Gibco 10270-106) in incubator at 37 °C in the presence of 5% CO_2_. Cell stocks were maintained in T75 flasks (Thermo Fisher Scientific) and passaging done twice a week using 0.25% trypsin (Gibco, 15090046 diluted in PBS-EDTA).

### 4.2 Proteomics and phosphoproteomics mass-spectrometry experiment

Proteomics experiments were performed using mass spectrometry as reported (Casado et al., 2013, Rajeeve et al., 2014). Urea lysis buffer was used to lyse the cells (8 M urea, 10 mM Na3VO4, 100 mM β-glycerol phosphate and 25 mM Na2H2P2O7 and supplemented with phosphatase inhibitors (Sigma)). Proteins were reduced and alkylated by addition of 1 mM DTT and 5 mM iodoacetamide, followed by overnight incubation with immobilised trypsin to digest proteins into peptides. Using OASIS HLB columns (Waters), in a vacuum manifold, peptides were desalted by solid phase extraction (SPE) following the manufacturer's guidelines apart that the elution buffer contained 1 M glycolic acid. Titanium dioxide (TiO_2_) enrichment beads (GL Sciences) was used for the phosphoproteomics analysis, similarly to that described (Casado et al., 2013, Montoya et al., 2011). Samples were analysed with LC-MS/MS using a LTQ-Orbitrap mass-spectrometer. Mascot was used to identify peptides against SwissProt human protein database, and Pescal used for quantification.

Nanoflow LC–MS/MS in an LTQ-orbitrap was used to analyse dried peptide extracts dissolved in 0.1% TFA. Gradient elution, 2–35%, from buffer B in 90 min with buffer A being used to balance the mobile phase (buffer A was 0.1% formic acid in water and B was 0.1% formic acid in acetonitrile). SwissProt (version 2013.03) was used to search the MS with mass window of 10 ppm and 600 mmu for parent and fragment mass to charge values. Only human entries were considered using Mascot search engine (version 2.38). Searches for variable modifications were constituted by oxidation of methionine, pyro-glu (N-term) and phosphorylation of serine, threonine and tyrosine. False discovery rate of less than 1%, calculated by comparing against decoy databases, was considered. Peak areas of the first three isotopes of each peptide ion extracted chromatographs (mass 7 ppm and 1.5 min retention time window) was used for quantification. Retention shifts were taken in consideration by re-calculating for each peptide in each LC–MS/MS run individually using linear regression based on common ions across runs. The mass spectrometry proteomics data have been deposited to the ProteomeXchange Consortium via the PRIDE (Vizcaíno et al., 2016) partner repository with the dataset identifier PXD006693.

### 4.3 Differential protein and phosphorylation analysis

MS intensities were log2 transformed followed by a scaling in each sample to account for potential pipetting differences. Only proteotypic peptides measured consistently across half of the replicates were considered. Differential protein and phosphorylation changes were performed between UOK262 and UOK262pFH, i.e. log2(UOK262) - log2(UOK262pFH). Statistically significant changes were estimated using independent *t*-test, followed by control for false discovery rate using Benjamini–Hochberg FDR.

### 4.4 Consumption and release quantification of metabolites

UOK262 and UOK262pFH were detached using trypsin as described above and counted using CASY Cell counter (OLS, Germany). Cells (1.5 × 10^5^) were plated onto 6-well plates (Thermo Fisher Scientific) and allowed to grow for 16 h. Cell culture media (200 µl) were collected from each well immediately after (t = 0). Then medium was replaced with fresh one and collected after additional 24 h of incubation. A plate with control medium only was prepared at t = 0 and collected after 24 h for background measurements. Cells from an additional plate prepared in parallel were used for counting or lysed in RIPA buffer for measurement of protein content at both t = 0 and t = 24. The collected media were centrifuged at 4 °C for 10 min at max speed and 50 µl of the supernatant extracted in 750 µl of cold metabolite extraction buffer (MEB) as previously described (Sciacovelli et al., 2016). The solution was centrifuged at 4 °C for 10 min at max speed and the supernatant was transferred onto LC-MS vials for metabolomics analyses. For protein content, RIPA extracts were measured using Pierce BCA Protein Assay kit (Thermo Fisher Scientific) following manufacturer's protocol.

LC-MS analysis of sample extracts was performed on a Q Exactive mass spectrometer coupled to Dionex UltiMate 3000 Rapid Separation LC system (Thermo Fisher Scientific). The liquid chromatography system was fitted with a SeQuant ZIC-pHILIC (150 mm × 2.1 mm, 5 µm) with guard column (20 mm × 2.1 mm, 5 µm) from Merck (Darmstadt, Germany). The mobile phase was composed of 20 mM ammonium carbonate and 0.1% ammonium hydroxide in water (solvent A), and acetonitrile (solvent B). The flow rate was set at 180 µl × min^−1^ with the following gradient: 0–1 min: hold at 70% B; 1–16 min: linear gradient from 70% to 38% B; 16–16.5 min: linear gradient from 38% to 70% B; 16.5–25 min: hold at 70% B. The mass spectrometer was operated in full MS and polarity switching mode. Medium from five independent cell cultures were analysed for each condition and samples were randomised in order to avoid bias in sample analyses due to machine drift. The acquired spectra were analysed using XCalibur Qual Browser and XCalibur Quan Browser software (Thermo Fisher Scientific) by referencing to an internal library of compounds.

Absolute quantification of metabolites in the cell culture medium was performed by interpolation of the corresponding standard curves obtained from commercially available compounds running with the same batch of samples. For each spent medium sample and each metabolite, the measured concentration spent was converted to consumption/release (CORE) data (molar amounts per dry weight per unit time) adapting the approach described in (Jain et al., 2012).

### 4.5 Immunohistochemistry on HLRCC human tumours

Specimens were formalin fixed and embedded in paraffin wax; 3-μm serial sections mounted on Snowcoat X-tra slides (Surgipath, Richmond, IL) were dewaxed in xylene and rehydrated using graded ethanol washes. For antigen retrieval, sections were immersed in preheated DAKO target retrieval solution (DAKO) and treated for 90 s in a pressure cooker. Sections analysed contained both tumour and adjacent normal renal parenchyma acting as an internal control; in addition, substitution of the primary antibody with antibody diluent was used as a negative control. Antigen/antibody complexes were detected using the Envision system (DAKO) according to the manufacturer's instructions, with the following modifications: incubation of primary antibody for 1 h in a humidified chamber. Sections were counterstained with Gill's haematoxylin for 30 s, dehydrated in graded ethanol washes, and mounted in DPX (Lamb, London, United Kingdom). Antibodies used were: anti-2-succinic-cysteine (2-SC, gift from Patrick Pollard, used 1:5000 dilution), PhosphoDetect™ Anti-PDH-E1α (pSer^2^^3^^2^) Rabbit pAb (used in 1:1000 dilution) purchased from Merck, and PDH Monoclonal Antibody (9H9AF5) (used in 1:200 dilution) purchased from ThermoFisher. All patients provided informed consent, REC approval number 16/WS/0039.

### 4.6 Metabolic extracts after glucose and glutamine labelling

UOK262 and UOK262pFH (1.5 × 10^5^) were plated onto 6-well plates (Thermo Fisher Scientific) and grown overnight. The day after, medium was replaced with fresh medium containing ^13^C_6_ glucose or ^13^C_5_ glutamine (Cambridge Isotope Laboratories). The following day, medium was extracted as described above at the indicated time points. Intracellular extracts were obtained as described before (Sciacovelli et al.2016). Cells were counted using Countess (Thermo Fisher Scientific) or CASY Cell counter (OLS, Germany) as described before (Sciacovelli et al., 2016).

### 4.7 Metabolic modelling using Recon 2.2

For this analysis the human metabolic reconstruction Recon 2.2 was used (Swainston et al., 2016). The commonly used ATP maintenance (ATPM) reaction was added to the model (ATP + H_2_O = > ADP + Phosphate + H^+^). This reaction accounts for ATP production not directly associated with biomass production. Two context-specific models were generated: one for UOK262 cells and another for UOK262pFH. UOK262 model contains the specific measured growth rate, 0.01973, and hard coded deletion of FH by setting the upper and lower bounds of reactions FUM and FUMm to zero. UOK262pFH model is only constrained with the measured growth rate, 0.02940, since FH expression and activity is restored in these cell lines the catalysed reactions were not constrained. Uptake rates for all metabolites are constrained to zero, apart of those metabolites present in cell culture medium, DMEM (Gibco 41966-029). For metabolites in the medium that were not measured or do not display significant CORE differences the exchange reactions lower bound were set similarly to the following publication (Yizhak et al., 2013). Consumption/Release (CORE) measurements were used to constrain the metabolic models of UOK262 and UOK262pFH, by constraining the respective exchange reaction rates. To avoid infeasible solutions due to measurement precision, the measured rates were fitted to the steady-state solution space using a linear implementation of minimization of metabolic adjustment (MOMA) (Segrè et al., 2002). Next, the models were simplified using flux variability analysis (FVA), thereby removing any reaction that is not capable of carrying flux. The models were then simulated using parsimonious flux balance analysis (pFBA) (Lewis et al., 2010). Since the growth rates are directly measured and used as model constraints, the simulations are performed by maximising the non-growth-associated ATP production (ATPM reaction).

### 4.8 Cell Lysates and western blot

UOK262 and UOK262pFH cells (6 × 10^5^) were plated onto 6-cm dishes. After 24 h, cells were washed twice in PBS on ice and then lysed using RIPA buffer. Protein content was quantified using Pierce BCA protein Assay (Thermo Fisher Scientific) following manufacturer's protocol. Lysed proteins (50–100 µg) were heated at 70 °C for 10 min in Bolt Loading Buffer 1x+4% β-mercapto-ethanol and then loaded onto a 4–12% Bolt Bis-Tris gel (Thermo Fisher Scientific). Gels were run at 165 V using Bolt MES1x buffer for 40 min. Dry transfer of the proteins onto nitrocellulose membrane was obtained using IBLOT2 (Thermo Fisher Scientific). Membrane was then blocked for 1 h at room temperature using in BSA or milk 5% in TBS 1X supplemented with Tween20 0.01% (TBST). Primary antibodies for Calnexin (1:2000, Abcam), V5 (1:5000,Thermo Fisher Scientific), PDH-E1a (1:500, Thermo Fisher Scientific), pSer^232^ PDH-E1a (1:500, Merck Millipore), and pSer^293^ PDH-E1a (1:500, Merck Millipore) were incubated overnight at 4 °C. The day after, the membrane was washed in TBST and then incubated with secondary antibodies for 1 h at room temperature (LiCOR, 1:2000, conjugated with 680 or 800 nm fluorophores). After washes in TBST, images were taken using Image Studio Lite software (LiCOR).

### 4.9 Code dependencies and availability

All the computational analysis were performed in Python version 2.7.10 and are available under GNU General Public License V3 as GitHub projects in the following url https://github.com/saezlab/hlrcc. Metabolic modelling and SBML import of Recon 2.2 was performed using python module Framed version 0.3.2 (Machado, 2017). Plotting was done using Python modules Matplotlib version 1.4.3 (Hunter, 2007) and Seaborn version 0.7.0 (Waskom et al., 2014). Python modules Scipy version 0.17.1 (Jones et al., 2016) and Numpy version 1.11.1 (der Walt et al., 2011) were used to perform efficient numerical calculations and statistical analysis. Biological data analysis and structuring was carried out using Python module Pandas version 0.18.1 (McKinney et al., 2010).

## References

[bib1] Beltrao P., Albanèse V., Kenner L.R., Swaney D.L., Burlingame A., Villén J., Lim W.A., Fraser J.S., Frydman J., Krogan N.J. (2012). Systematic functional prioritization of protein posttranslational modifications. Cell.

[bib2] Casado P., Rodriguez-Prados J.-C., Cosulich S.C., Guichard S., Vanhaesebroeck B., Joel S., Cutillas P.R. (2013). Kinase-substrate enrichment analysis provides insights into the heterogeneity of signaling pathway activation in leukemia cells. Sci. Signal..

[bib3] der Walt S. van, Colbert S.C., Varoquaux G. (2011). The NumPy array: a structure for efficient numerical computation. Comput. Sci. Eng..

[bib4] Dinkel H., Chica C., Via A., Gould C.M., Jensen L.J., Gibson T.J., Diella F. (2011). Phospho.ELM: a database of phosphorylation sites—update 2011. Nucleic Acids Res..

[bib5] Duarte N.C., Becker S.A., Jamshidi N., Thiele I., Mo M.L., Vo T.D., Srivas R., Palsson B.Ø. (2007). Global reconstruction of the human metabolic network based on genomic and bibliomic data. Proc. Natl. Acad. Sci. USA.

[bib6] Frezza C., Zheng L., Folger O., Rajagopalan K.N., MacKenzie E.D., Jerby L., Micaroni M., Chaneton B., Adam J., Hedley A., Kalna G., Tomlinson I.P.M., Pollard P.J., Watson D.G., Deberardinis R.J., Shlomi T., Ruppin E., Gottlieb E. (2011). Haem oxygenase is synthetically lethal with the tumour suppressor fumarate hydratase. Nature.

[bib7] Gaude E., Frezza C. (2016). Tissue-specific and convergent metabolic transformation of cancer correlates with metastatic potential and patient survival. Nat. Commun..

[bib8] Gonçalves E., Raguz Nakic Z., Zampieri M., Wagih O., Ochoa D., Sauer U., Beltrao P., Saez-Rodriguez J. (2017). Systematic analysis of transcriptional and post-transcriptional regulation of metabolism in yeast. PLoS Comput. Biol..

[bib9] Grant C.M. (2008). Metabolic reconfiguration is a regulated response to oxidative stress. J. Biol..

[bib10] Hanahan D., Weinberg R.A. (2011). Hallmarks of cancer: the next generation. Cell.

[bib11] Hanahan D., Weinberg R.A. (2000). The hallmarks of cancer. Cell.

[bib12] Hornbeck P.V., Zhang B., Murray B., Kornhauser J.M., Latham V., Skrzypek E. (2015). PhosphoSitePlus, 2014: mutations, PTMs and recalibrations. Nucleic Acids Res..

[bib13] Hu J., Locasale J.W., Bielas J.H., O’Sullivan J., Sheahan K., Cantley L.C., Vander Heiden M.G., Vitkup D. (2013). Heterogeneity of tumor-induced gene expression changes in the human metabolic network. Nat. Biotechnol..

[bib14] Hunter J.D. (2007). Matplotlib: a 2D graphics environment. Comput. Sci. Eng..

[bib15] Isaacs J.S., Jung Y.J., Mole D.R., Lee S., Torres-Cabala C., Chung Y.-L., Merino M., Trepel J., Zbar B., Toro J., Ratcliffe P.J., Linehan W.M., Neckers L. (2005). HIF overexpression correlates with biallelic loss of fumarate hydratase in renal cancer: novel role of fumarate in regulation of HIF stability. Cancer Cell.

[bib16] Jain M., Nilsson R., Sharma S., Madhusudhan N., Kitami T., Souza A.L., Kafri R., Kirschner M.W., Clish C.B., Mootha V.K. (2012). Metabolite profiling identifies a key role for glycine in rapid cancer cell proliferation. Science.

[bib17] Jones, E., Oliphant, T., Peterson, P., et al., 2016. SciPy: Open source scientific tools for Python.

[bib18] Kato M., Wynn R.M., Chuang J.L., Tso S.-C., Machius M., Li J., Chuang D.T. (2008). Structural basis for inactivation of the human pyruvate dehydrogenase complex by phosphorylation: role of disordered phosphorylation loops. Structure.

[bib19] Korotchkina L.G., Patel M.S. (2001). Site specificity of four pyruvate dehydrogenase kinase isoenzymes toward the three phosphorylation sites of human pyruvate dehydrogenase. J. Biol. Chem..

[bib20] Lewis N.E., Hixson K.K., Conrad T.M., Lerman J.A., Charusanti P., Polpitiya A.D., Adkins J.N., Schramm G., Purvine S.O., Lopez-Ferrer D., Weitz K.K., Eils R., König R., Smith R.D., Palsson B.Ø. (2010). Omic data from evolved E. coli are consistent with computed optimal growth from genome-scale models. Mol. Syst. Biol..

[bib21] Machado, D., 2017. Framed.

[bib22] Machado D., Herrgård M. (2014). Systematic evaluation of methods for integration of transcriptomic data into constraint-based models of metabolism. PLoS Comput. Biol..

[bib23] McKinney, W., et al., 2010. Data structures for statistical computing in python. In: Proceedings of the 9th Python in Science Conference. pp. 51–56.

[bib24] Mertins P., Mani D.R., Ruggles K.V., Gillette M.A., Clauser K.R., Wang P., Wang X., Qiao J.W., Cao S., Petralia F., Kawaler E., Mundt F., Krug K., Tu Z., Lei J.T., Gatza M.L., Wilkerson M., Perou C.M., Yellapantula V., Huang K.-L., Lin C., McLellan M.D., Yan P., Davies S.R., Townsend R.R., Skates S.J., Wang J., Zhang B., Kinsinger C.R., Mesri M., Rodriguez H., Ding L., Paulovich A.G., Fenyö D., Ellis M.J., Carr S.A., CPTAC N.C.I. (2016). Proteogenomics connects somatic mutations to signalling in breast cancer. Nature.

[bib25] Montoya A., Beltran L., Casado P., Rodríguez-Prados J.-C., Cutillas P.R. (2011). Characterization of a TiO_2_ enrichment method for label-free quantitative phosphoproteomics. Methods.

[bib26] Oliveira A.P., Ludwig C., Zampieri M., Weisser H., Aebersold R., Sauer U. (2015). Dynamic phosphoproteomics reveals TORC1-dependent regulation of yeast nucleotide and amino acid biosynthesis. Sci. Signal..

[bib27] Raguz Nakic Z., Seisenbacher G., Posas F., Sauer U. (2016). Untargeted metabolomics unravels functionalities of phosphorylation sites in Saccharomyces cerevisiae. BMC Syst. Biol..

[bib28] Rajeeve V., Vendrell I., Wilkes E., Torbett N., Cutillas P.R. (2014). Cross-species proteomics reveals specific modulation of signaling in cancer and stromal cells by phosphoinositide 3-kinase (PI3K) inhibitors. Mol. Cell. Proteom..

[bib29] Sciacovelli M., Gonçalves E., Johnson T.I., Zecchini V.R., da Costa A.S.H., Gaude E., Drubbel A.V., Theobald S.J., Abbo S.R., Tran M.G.B., Rajeeve V., Cardaci S., Foster S., Yun H., Cutillas P., Warren A., Gnanapragasam V., Gottlieb E., Franze K., Huntly B., Maher E.R., Maxwell P.H., Saez-Rodriguez J., Frezza C. (2016). Fumarate is an epigenetic modifier that elicits epithelial-to-mesenchymal transition. Nature.

[bib30] Segrè D., Vitkup D., Church G.M. (2002). Analysis of optimality in natural and perturbed metabolic networks. Proc. Natl. Acad. Sci. USA.

[bib31] Seifert F., Ciszak E., Korotchkina L., Golbik R., Spinka M., Dominiak P., Sidhu S., Brauer J., Patel M.S., Tittmann K. (2007). Phosphorylation of serine 264 impedes active site accessibility in the E1 component of the human pyruvate dehydrogenase multienzyme complex. Biochemistry.

[bib32] Sourbier C., Ricketts C.J., Matsumoto S., Crooks D.R., Liao P.-J., Mannes P.Z., Yang Y., Wei M.-H., Srivastava G., Ghosh S., Chen V., Vocke C.D., Merino M., Srinivasan R., Krishna M.C., Mitchell J.B., Pendergast A.M., Rouault T.A., Neckers L., Linehan W.M. (2014). Targeting ABL1-mediated oxidative stress adaptation in fumarate hydratase-deficient cancer. Cancer Cell.

[bib33] Stratton M.R., Campbell P.J., Futreal P.A. (2009). The cancer genome. Nature.

[bib34] Subramanian A., Tamayo P., Mootha V.K., Mukherjee S., Ebert B.L., Gillette M.A., Paulovich A., Pomeroy S.L., Golub T.R., Lander E.S., Mesirov J.P. (2005). Gene set enrichment analysis: a knowledge-based approach for interpreting genome-wide expression profiles. Proc. Natl. Acad. Sci. USA.

[bib35] Swainston N., Smallbone K., Hefzi H., Dobson P.D., Brewer J., Hanscho M., Zielinski D.C., Ang K.S., Gardiner N.J., Gutierrez J.M., Kyriakopoulos S., Lakshmanan M., Li S., Liu J.K., Martínez V.S., Orellana C.A., Quek L.-E., Thomas A., Zanghellini J., Borth N., Lee D.-Y., Nielsen L.K., Kell D.B., Lewis N.E., Mendes P. (2016). Recon 2.2: from reconstruction to model of human metabolism. Metabolomics.

[bib36] The Gene Ontology Consortium (2015). Gene ontology consortium: going forward. Nucleic Acids Res..

[bib37] Thiele I., Swainston N., Fleming R.M.T., Hoppe A., Sahoo S., Aurich M.K., Haraldsdottir H., Mo M.L., Rolfsson O., Stobbe M.D., Thorleifsson S.G., Agren R., Bölling C., Bordel S., Chavali A.K., Dobson P., Dunn W.B., Endler L., Hala D., Hucka M., Hull D., Jameson D., Jamshidi N., Jonsson J.J., Juty N., Keating S., Nookaew I., Le Novère N., Malys N., Mazein A., Papin J.A., Price N.D., Selkov E., Sigurdsson M.I., Simeonidis E., Sonnenschein N., Smallbone K., Sorokin A., van Beek J.H.G.M., Weichart D., Goryanin I., Nielsen J., Westerhoff H.V., Kell D.B., Mendes P., Palsson B.Ø. (2013). A community-driven global reconstruction of human metabolism. Nat. Biotechnol..

[bib38] Tomlinson I.P.M., Alam N.A., Rowan A.J., Barclay E., Jaeger E.E.M., Kelsell D., Leigh I., Gorman P., Lamlum H., Rahman S., Roylance R.R., Olpin S., Bevan S., Barker K., Hearle N., Houlston R.S., Kiuru M., Lehtonen R., Karhu A., Vilkki S., Laiho P., Eklund C., Vierimaa O., Aittomäki K., Hietala M., Sistonen P., Paetau A., Salovaara R., Herva R., Launonen V., Aaltonen L.A., Multiple Leiomyoma Consortium (2002). Germline mutations in FH predispose to dominantly inherited uterine fibroids, skin leiomyomata and papillary renal cell cancer. Nat. Genet..

[bib39] Tyrakis P.A., Yurkovich M.E., Sciacovelli M., Papachristou E.K., Bridges H.R., Gaude E., Schreiner A., D’Santos C., Hirst J., Hernandez-Fernaud J., Springett R., Griffiths J.R., Frezza C. (2017). Fumarate Hydratase Loss Causes Combined Respiratory Chain Defects: Cell Rep.

[bib40] Vizcaíno J.A., Csordas A., del-Toro N., Dianes J.A., Griss J., Lavidas I., Mayer G., Perez-Riverol Y., Reisinger F., Ternent T., Xu Q.-W., Wang R., Hermjakob H. (2016). 2016 update of the PRIDE database and its related tools. Nucleic Acids Res..

[bib41] Waskom, M., Botvinnik, O., Hobson, P., Cole, J.B., Halchenko, Y., Hoyer, S., Miles, A., Augspurger, T., Yarkoni, T., Megies, T., Coelho, L.P., Wehner, D., cynddl, Ziegler, E., diego, Zaytsev, Y.V., Hoppe, T., Seabold, S., Cloud, P., Koskinen, M., Meyer, K., Qalieh, A., Allan, D., 2014. Seaborn: v0.5. 0 (November 2014). ZENODO. doi: 〈http://doi.org/10.5281/zenodo.12710〉.

[bib42] Yang M., Ternette N., Su H., Dabiri R., Kessler B.M., Adam J., Teh B.T., Pollard P.J. (2014). The Succinated proteome of FH-mutant tumours. Metabolites.

[bib43] Yang Y., Lane A.N., Ricketts C.J., Sourbier C., Wei M.-H., Shuch B., Pike L., Wu M., Rouault T.A., Boros L.G., Fan T.W.-M., Linehan W.M. (2013). Metabolic reprogramming for producing energy and reducing power in fumarate hydratase null cells from hereditary leiomyomatosis renal cell carcinoma. PLoS One.

[bib44] Yizhak K., Gabay O., Cohen H., Ruppin E. (2013). Model-based identification of drug targets that revert disrupted metabolism and its application to ageing. Nat. Commun..

[bib45] Zhang H., Liu T., Zhang Z., Payne S.H., Zhang B., McDermott J.E., Zhou J.-Y., Petyuk V.A., Chen L., Ray D., Sun S., Yang F., Chen L., Wang J., Shah P., Cha S.W., Aiyetan P., Woo S., Tian Y., Gritsenko M.A., Clauss T.R., Choi C., Monroe M.E., Thomas S., Nie S., Wu C., Moore R.J., Yu K.-H., Tabb D.L., Fenyö D., Bafna V., Wang Y., Rodriguez H., Boja E.S., Hiltke T., Rivers R.C., Sokoll L., Zhu H., Shih I.-M., Cope L., Pandey A., Zhang B., Snyder M.P., Levine D.A., Smith R.D., Chan D.W., Rodland K.D., CPTAC Investigators (2016). Integrated proteogenomic characterization of human high-grade serous ovarian cancer. Cell.

[bib46] Zheng L., Mackenzie E.D., Karim S.A., Hedley A., Blyth K., Kalna G., Watson D.G., Szlosarek P., Frezza C., Gottlieb E. (2013). Reversed argininosuccinate lyase activity in fumarate hydratase-deficient cancer cells. Cancer Metab..

