# Corrigendum to “Post-translational regulation of metabolism in fumarate hydratase deficient cancer cells” [Metabol. Eng. 45 (2018) 149–157]

**DOI:** 10.1016/j.ymben.2024.01.002

**Published:** 2024-03

**Authors:** Emanuel Gonçalves, Marco Sciacovelli, Ana S.H. Costa, Maxine Gia Binh Tran, Timothy Isaac Johnson, Daniel Machado, Christian Frezza, Julio Saez-Rodriguez

**Affiliations:** a European Molecular Biology Laboratory, European Bioinformatics Institute, EMBL-EBI, Wellcome Genome Campus, Cambridge, CB10 1SD, UK; b Medical Research Council Cancer Unit, University of Cambridge, Cambridge, CB2 0XZ, UK; c European Molecular Biology Laboratory, EMBL, Heidelberg, Germany; d UCL Division of Surgery and Interventional Science, Specialist Center for Kidney Cancer, Royal Free Hospital, Pond Street, London, NW3 2QG, UK; e RWTH Aachen University, Faculty of Medicine, Joint Research Center for Computational Biomedicine, Aachen, Germany; f Centre of Biological Engineering, University of Minho, Braga, Portugal

The authors regret to inform the readers that the 2SC staining for the normal kidney of Patient 1 in Fig. 4C was an unintentional duplication of the PDH-E1a-pSer232 staining of the same patient that occurred during the final assembly of the figure. The correct panel is now included in this Corrigendum. We thank the anonymous reader in Pubpeer for pointing this mistake out.

**Figure:**
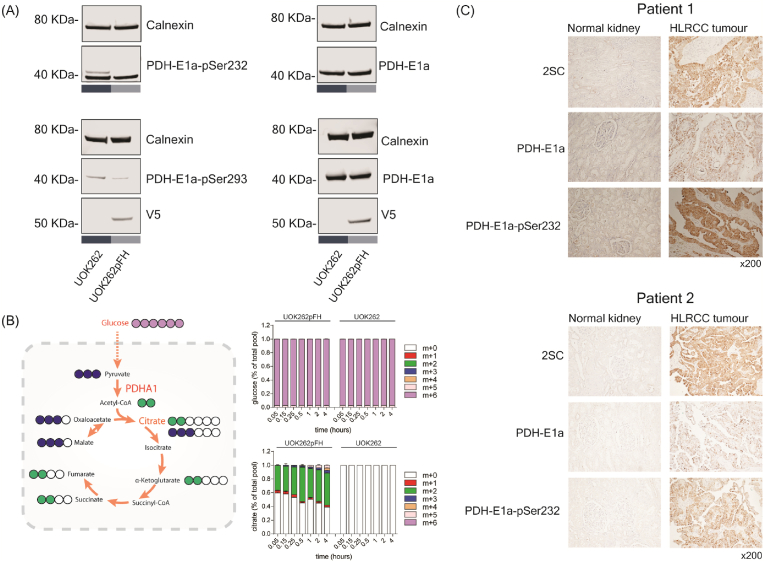

The authors would like to apologise for any inconvenience caused.

