# Supplementary material for: Post-translational regulation of metabolism in fumarate hydratase deficient cancer cells
Source: Metab Eng. 2018 Jan;45:149–57. doi: 10.1016/j.ymben.2017.11.011 (PMC5805855; doi:10.1016/j.ymben.2017.11.011)
Supplement: Supplementary file 1 — Supplementary material [file mmc1.docx]

### **Supplementary figures**

**
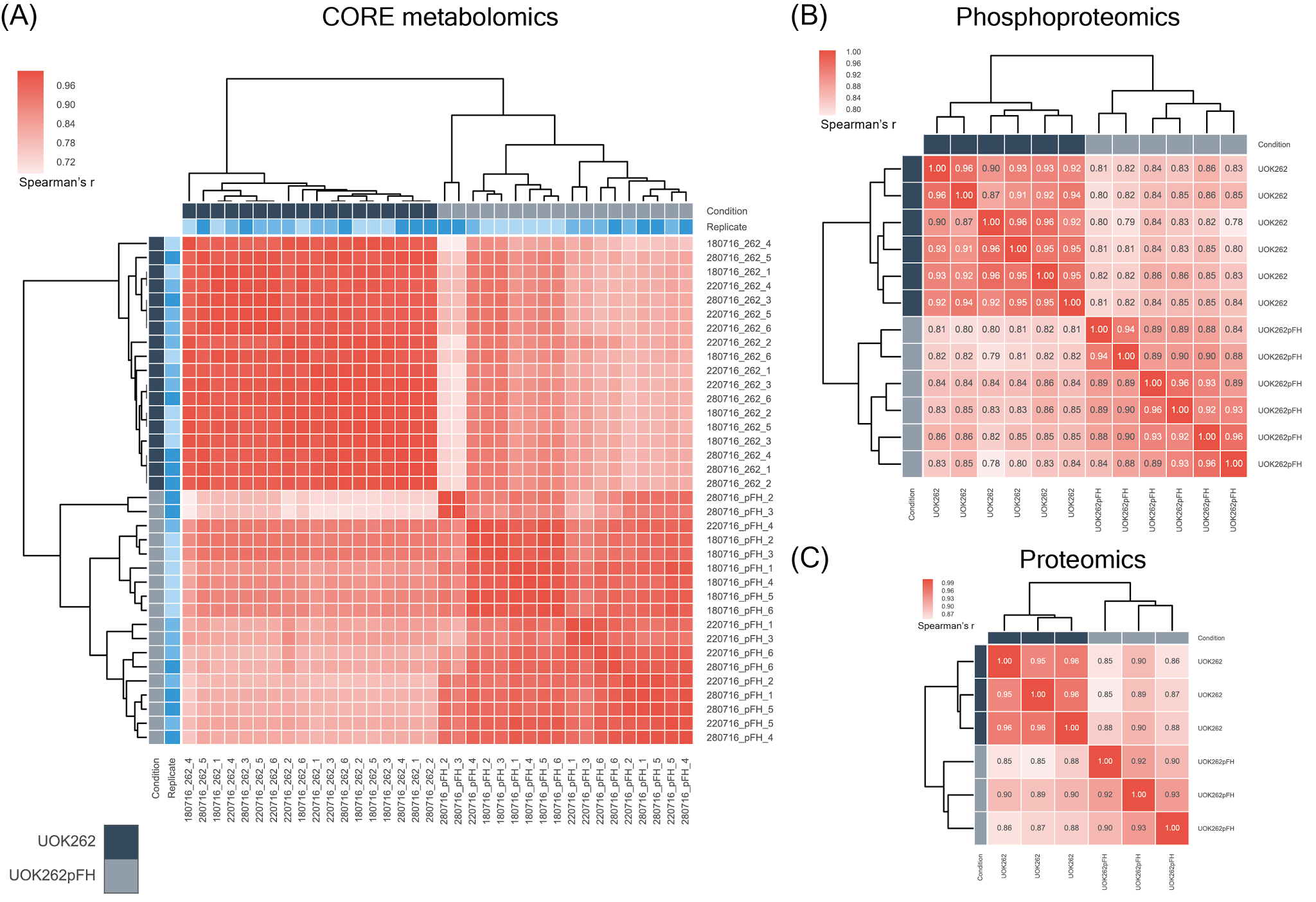
**

**Supplementary Figure 1.** Unsupervised clustering of the experimental replicates. A) Clustering analysis of three different biological and technical replicates of the CORE metabolomics experiments. Light blue colour denotes the different biological replicates. B) Clustering of the three biological replicates, where each has a technical replicate, of the phosphoproteomics experiments. C) Clustering analysis of the three biological replicates of the proteomics measurements.

**
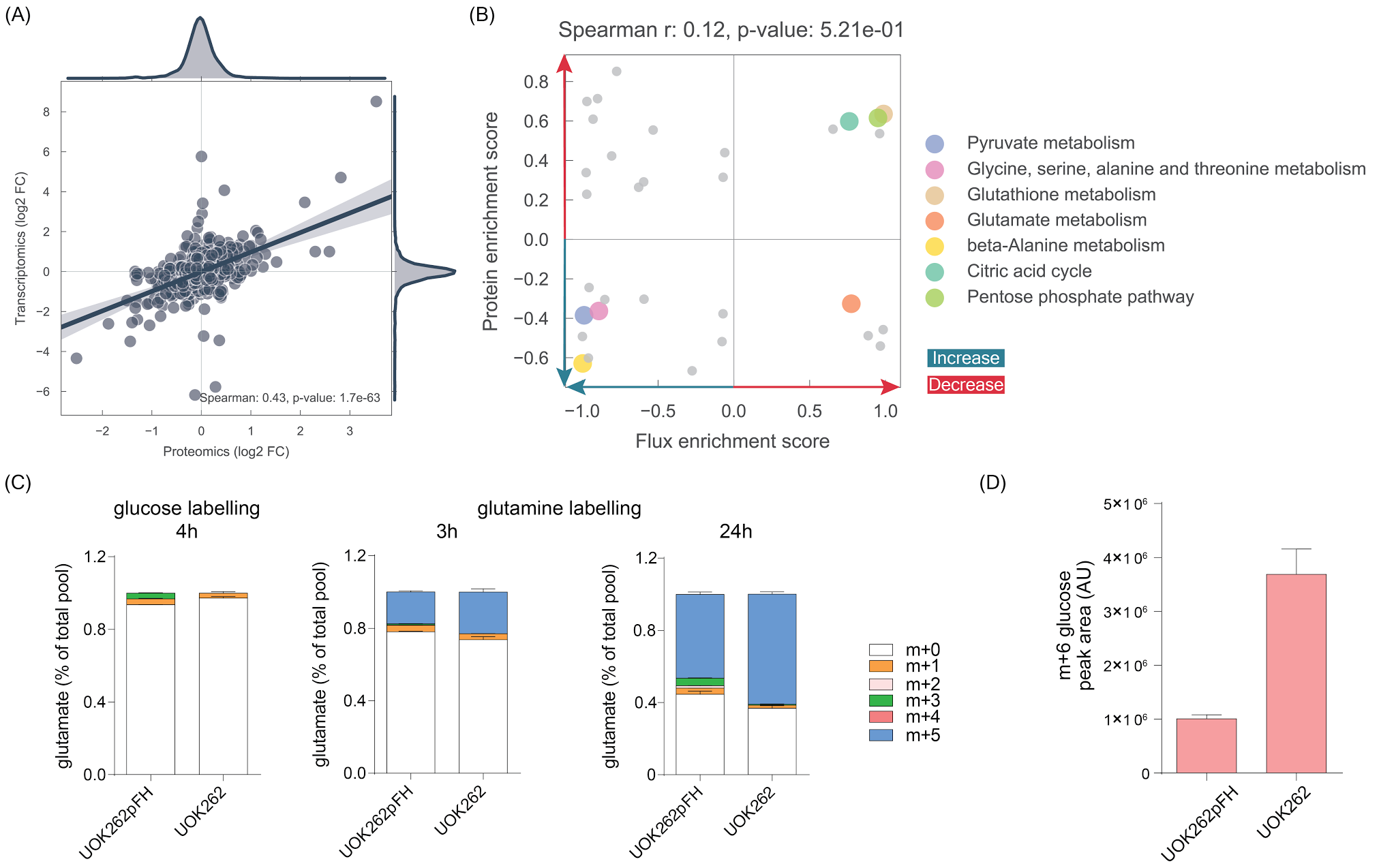
**

**Supplementary Figure 2.** Proteomics correlation analysis. A) Correlation between proteomics and transcriptomics measurements. Log2 fold changes between UOK262 and UOK262pFH were used. B) Scatter plot with the GSEA enrichment score obtained for each metabolic pathway in the metabolic model for which it was possible to overlap at least 5 measured proteins or *in silico* estimated fluxes. Representative pathways were highlighted. C) Glutamate labelling from either glucose or glutamine, secreted in the medium of UOK262 and UOK262pFH. The time point used for glucose labelling is 4 h while for glutamine is 3 h and 24h. D) Glucose uptake in UOK262pFH and UOK262.

### **Tables**

| **Table 1.** Metabolic enzymes differentially phosphorylated sites. | | | | |
| --- | --- | --- | --- | --- |
| **Enzyme** | **Phosphosite** | **Fold-change** | **p-value** | **FDR** |
| GAPDH | S83 | -0.93 | 7.9E-05 | 9.3E-04 |
| HMGCS1 | S495 | -0.81 | 7.3E-07 | 3.3E-05 |
| CTPS1 | S575 | -0.70 | 1.4E-04 | 1.4E-03 |
| MTMR3 | S613 | -0.52 | 5.8E-05 | 7.2E-04 |
| CTPS1 | S574 | -0.47 | 4.5E-05 | 6.2E-04 |
| PGK1 | S203 | -0.40 | 5.2E-05 | 6.7E-04 |
| DPYSL3 | T509 | -0.39 | 5.5E-05 | 6.9E-04 |
| PGM1 | S117 | -0.39 | 1.8E-04 | 1.6E-03 |
| IMPDH1 | S160 | -0.37 | 3.3E-03 | 1.3E-02 |
| IMPDH2 | S160 | -0.37 | 3.3E-03 | 1.3E-02 |
| PIK3C2A | S884 | -0.36 | 5.2E-04 | 3.4E-03 |
| DPYSL3 | S522 | -0.36 | 8.1E-05 | 9.3E-04 |
| PI4KB | S428 | -0.34 | 4.4E-04 | 3.0E-03 |
| PGM3 | T62 | -0.16 | 7.9E-03 | 2.6E-02 |
| PCYT1A | S315 | 0.37 | 1.9E-04 | 1.7E-03 |
| PCYT1B | S315 | 0.37 | 1.9E-04 | 1.7E-03 |
| BCKDHA | S347 | 0.41 | 1.5E-03 | 7.6E-03 |
| NAA10 | S205 | 0.46 | 4.3E-03 | 1.6E-02 |
| RRM2 | S20 | 0.47 | 1.8E-04 | 1.6E-03 |
| GUCY1B2 | S150 | 0.52 | 4.4E-03 | 1.6E-02 |
| PDHA1 | S232 | 0.52 | 9.8E-06 | 2.3E-04 |
| CMPK1 | S180 | 0.67 | 7.4E-03 | 2.5E-02 |
| BCKDHA | S337 | 0.73 | 2.5E-03 | 1.1E-02 |

### **Supplementary Tables**

**Supplementary Table 1.** Differential analysis of proteomics, phosphoproteomics and CORE metabolomics.

**Supplementary Table 2.** List of *in silico* estimated fluxes for UOK262 and UOK262pFH cells.

**Supplementary Table 3.** GSEA enrichment analysis output performed in both proteomics and fluxomics data-sets.

**Supplementary Table 4.** List of putative regulatory phosphorylation-sites located in metabolic enzymes.
